# COVID-19 pandemic in Bangladesh: A scoping review of governance issues affecting response in public sector

**DOI:** 10.1016/j.puhip.2023.100457

**Published:** 2023-12-16

**Authors:** Syed Masud Ahmed, Mahruba Khanam, Noshin Sayiara Shuchi

**Affiliations:** a Centre of Excellence for Health Systems and Universal Health Coverage (CoE HS&UHC), BRAC James P Grant School of Public Health, BRAC University, Bangladesh; b Bangladesh Health Watch, BRAC James P Grant School of Public Health, BRAC University, Dhaka, Bangladesh; c Health Systems and Population Studies Division, International Centre for Diarrhoeal Disease Research, Bangladesh

**Keywords:** COVID-19 pandemic, Health system, Governance, Bangladesh, Health-sector corruption

## Abstract

**Background:**

On January 30, 2020, WHO declared COVID-19 as a Global Public Health Emergency. The first three COVID-19 cases in Bangladesh were confirmed on March 8, 2020. Thus, Bangladesh got substantial time to prepare the people and the health systems to respond to the outbreak However, neither the health ministry nor the government was found to rise to the occasion and provide the necessary stewardship for a coordinated and comprehensive response.

**Objective:**

The importance of governance to mount an evidence-based pandemic response cannot be overemphasised. This study presents critical reflections on the Bangladesh government's COVID-19 response through a review of selected papers, with expert deliberations on the review findings to consolidate emerging lessons for future pandemic preparedness.

**Study design:**

A scoping review approach was taken for this study.

**Methods:**

Documents focusing on COVID-19 governance were selected from a repository of peer-reviewed articles published by researchers using data from Bangladesh (n = 11).

**Results:**

Findings reveal Bangladesh's COVID-19 response to be delayed, slow, and ambiguous, reflecting poorly on its governance. Lack of governance capability in screening for COVID-19, instituting quarantine and lockdown measures in the early weeks, safety and security of frontline healthcare providers, timely and equitable COVID-19 testing, and logistics and procurement were phenomenal. The pandemic unmasked the weaknesses of the health system in this regard and “created new opportunities for corruption.” The failure to harmonise coordination among the government's different agencies for the COVID-19 response, along with poor risk communication, which was not culture-sensitive and context-specific. Over time, the government initiated necessary actions to mitigate the pandemic's impact on the lives and livelihoods of the people. Diagnostic and case management services gained strength after some initial faltering; however, the stewardship functions were not seamless.

**Conclusions:**

Shortage of healthcare workers, incapability of health facilities to cater to COVID-19 suspects and cases, absence of health system resilience, and corruption in procurement and purchases were limited the government's COVID-19 response. These need urgent attention from policymakers to better prepare for the next epidemic/pandemic.

## 1 Introduction

The SARS-CoV-2 virus responsible for the COVID-19 pandemic first appeared in December 2019 in Wuhan, China [1]. It caught the world's attention in total surprise, unlike any past epidemic/pandemic, e.g., the Great Influenza pandemic during World War I [2]. Beginning in 2020, news of the outbreak of COVID-19 disease started to flash around the world, including Bangladesh. On January 30, 2020, WHO declared COVID-19 as a Global Public Health Emergency [3] and a pandemic on March 11, 2020. After the initial inertia, scientists worldwide joined hands to investigate various dimensions of the disease to mount an evidence-based pandemic response to contain morbidity and mortality. An explosive state of publications occurred to fulfil the urge to share covid data early for critical and strategic decision-making for prevention and treatment [4,5]. *Science* termed this one of “the biggest explosions of scientific literature ever” [6]. Interestingly, Bangladesh also joined this race to generate and disseminate new knowledge on COVID-19 although the culture of research and publication still has much to offer [7,8]. The beliefs, attitudes, and conventions within the academic community have previously demonstrated a lack of attention to research and a lack of proper financing, which needs to further evolve for translating evidence into policies [8]

The first three COVID-19 cases in Bangladesh were confirmed on March 8, 2020 [9]. Thus, Bangladesh got a valuable lead time of around five weeks (seven weeks if we count the last two weeks of January) to prepare the people and the health systems to respond to the outbreak, including the impending surge of patients in healthcare facilities. However, neither the health ministry nor the government was found to rise to the occasion and provide the necessary stewardship for a coordinated and comprehensive response. The political establishment followed a ‘go alone’ and ‘reactive’ approach relying on the bureaucracy and was mostly indifferent to the advice of public health scientists and practitioners. There were also severe flaws in the ‘transparency and accountability of the government's various pandemic-related responses, such as emergency procurement and purchases, and corruption was all-embracing [[10], [11], [12]].

Since the pandemic's beginning, Bangladeshi researchers and academics at home and abroad have begun to conduct and publish rapid studies on issues of immediate importance to feed the policymakers for appropriate COVID-19 response [13]. However, these publications, using data originating from Bangladesh by authors working in Bangladeshi institutions are dispersed widely in various data sources, and it is not easy for researchers and stakeholders to trace and find these for various academic and real-time use in policy and practice. This situation motivated Bangladesh Health Watch, a civil society watchdog on health issues, to archive these publications on COVID-19 in Bangladesh into an online research repository to facilitate this process, and covered a wide variety of themes and topics of importance [14]. This study reviewed and analysed selected papers on governance issues related to the COVID-19 pandemic in Bangladesh and attempted to consolidate emerging lessons for effective and evidence-based epidemics/pandemic response in future.

## 2 Methods

### 2.1 Settings

Bangladesh Health Watch ((https://bangladeshhealthwatch.org) is a civil society initiative for advocacy and monitoring to improve Bangladesh's health system by critically reviewing policies and programmes and facilitating appropriate actions for change. BHW has taken the initiative to archive all peer-reviewed publications on COVID-19 by Bangladeshi authors from home and abroad in a “research repository” that used data originating from Bangladesh (https://r.bangladeshhealthwatch.org). The idea was to make it easy for policymakers and practitioners, academics and researchers, and stakeholders within and outside Bangladesh to search and retrieve COVID-19-related documents for necessary use.

A scoping review approach retrieved relevant documents on COVID-19 governance (seeTable 1) following a protocol which defined key search terms, search engines to use, the range of documents to search, and the search period (see Table 2).

**Table 1:** Operational definitions of key terms used in the search protocol.

Key terms	Definition
COVID-19	An infectious disease caused by the SARS- CoV-2 virus that causes mild to severe respiratory illness to people and spreads mainly through respiratory droplets/aerosols, causing both short-term and long-lasting complications [[15], [16], [17]].
Governance	Governance is the structure and processes in which power is exercised to manage a country's economic and social resources for development. It is designed to ensure transparency, accountability, and responsiveness [18,19].

**Table 2:** The search protocol used for retrieving documents from the ‘research repository'.

Research Questions	i) What and how quarantine measures were taken; ii) What measures were taken for screening of suspected cases for COVID-19; What measures were taken to contain corruption in the health system; What measures were taken to procure relevant materials (masks, protective gears, disinfectants etc.) to contain COVID-19	
Objectives	To study governance of issues related to quarantine, screening, corruption and procurement and consolidate lessons learned for future evidence-based epidemic/pandemic response	
Search strategy	Inclusion criteria	Articles focusing on responses of COVID-19 pandemic in Bangladesh
		Full-text peer-reviewed journal articles
		Language: English
		Author(s): At least one author from Bangladeshi Institution
		Data origin: Bangladesh
	Exclusion criteria	Countries other than Bangladesh

		Articles published beyond timeframe, not full text
	Time frame	Articles published between March 2020–June 2021
Data source	Electronic database	PubMed, LitCovid and Scopus
	Institutional websites	Bangladesh Health Watch COVID-19 research repository

Search terms	
Category	Specific search terms
Public health	Health OR Mental health OR Health system OR Frontline health workforce OR Prevention OR Treatment OR Vaccine
COVID-19	Coronavirus OR COVID-19 OR COVID-19 pandemic
Governance	Response preparedness OR Management OR Capacity OR Challenges
Geographical context	Data originating from Bangladesh

The search terms were used in different combinations. The Boolean operators' AND’ & ‘OR’ were used to connect these search terms in order to find the most suitable articles.

### 2.2 Selection process

**Fig. 1:**
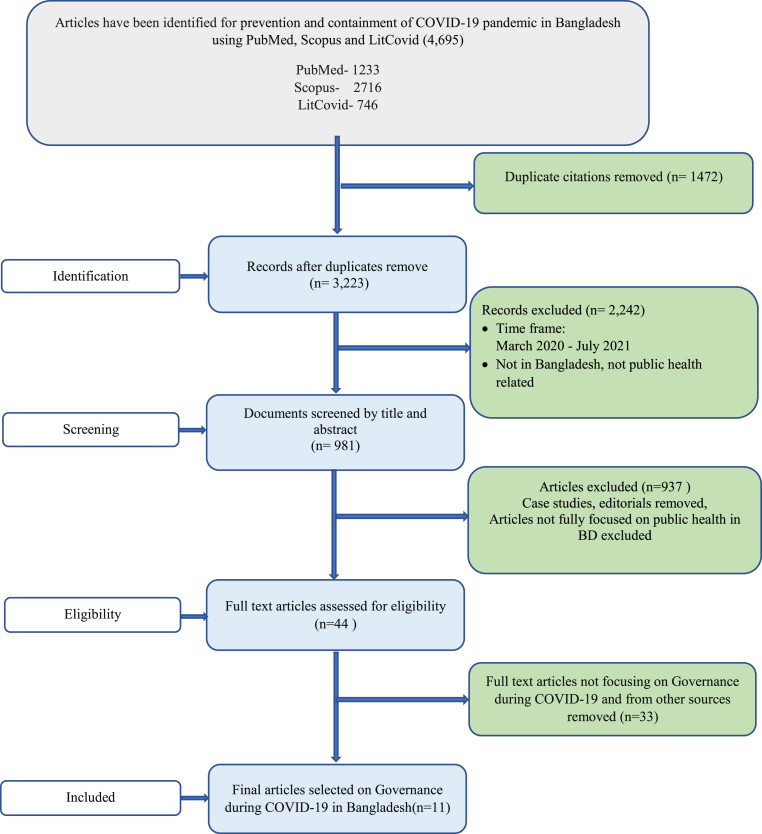
PRISMA diagram showing the selection process of the peer-reviewed articles.

After identifying all articles on COVID-19 in Bangladesh fulfilling the above conditions, duplicates were removed, and the titles and abstracts were screened for relevancy. A second stage screening was done to remove non-peer-reviewed articles, articles that were not full text, and articles that were beyond the timeframe. One reviewer (NSS) searched and screened the selected databases for extracting relevant articles. A second reviewer (MK) check was done for the quality assessment of included articles. After discussion, the two reviewers resolved any confusion or disagreement at the screening and data extraction stage. Any further confusion was addressed with support from the lead author. Articles meeting all the inclusion criteria were finally selected for analysis (n = 11) (see Fig. 1).

### 2.3 Data extraction and analysis

Data were extracted using a pre-designed template that included columns for the name of authors, theme/sub-themes, codes used, study population and key findings. From the wide range of data gather from the “research repository”, we identified and used the data related to ‘governance’ for this study. We dida descriptive analysis of the selected articles. We developed a brief narrative synthesis, specifically to inform an invited expert who critically reflected on the review findings, discussed these in the context of other Asian LMICs and identified key take-home messages. As we worked with secondary data, no ethical approval was obtained for this step. Finally, recommendations were made for future research in specific areas to develop the relevant topic further.

### 2.4 Expert deliberations

The review findings on the governance of Bangladesh's COVID-19 response were shared with some invited experts with varying backgrounds in public health, governance, and pandemic response in a deliberative discussion. They critically reflected on the findings and discussed at length the its importnance and significance in the context of Bangladesh. All the experts pointed out the governance shortcomings in taking quarantine measures, implementing screening for COVID-19, mitigating corruption and logistics of procurement. The findings from the reviewed articles are presented in Table 3.

**Table 3:** Summary of selected articles for this study.

Sl.	Title	Author & Year	Study setting and sample	Design/Type of Study	Key findings
1	Population-Level Preparedness About Preventive Practices Against Coronavirus Disease 2019: A Cross-Sectional Study Amon Adults in Bangladesh	Hossain et al., 2021	**Study Area and Sample:** All over Bangladesh among 1056 people, aged 18 years and above **Time frame:** 10–16 May 2020	Cross-sectional study	• Overall preparedness level was 68.9%, economic was 6.49 • Urban population had an advantage • Financial urgency was a barrier to people following the social distancing guidelines. • Community level preparedness had an impact on people's behavioural practices
2	COVID-19 in Bangladesh: Data deficiency to delayed decision	Huq & Biswas 2020	**Study Area and Sample:** All over Bangladesh	Viewpoint	• Several crises emerged starting from lack of testing facilities and a shortage of disinfectants and PPE. Country-wide fear started due to lack of timely initiatives by the government. • Number of tests performed were very few compared to population, absence of careful planning, dual burden of transmission risk.
3	Urban educated group's perceptions of the COVID-19 pandemic management in Bangladesh: a qualitative exploration	Joarder et al., 2021	**Study Area and Sample:** Bangladeshi urban population; 7 FGDs among purposely selected participants from an online survey **Time frame:** 19 May; 15 & Jun. 17, 2020	Qualitative study	• Failure in decision making, not engaging right people, lack of coordination resulted in a public health crisis and rapid spread of the disease in certain areas. • Failure in providing families sufficient time to prepare for lockdown and no allowance was provided to the poor • Initially critical times were wasted by testing 4 at a single testing facility • Corruption with PPE, equipment and mismanagement of relief materials, along with miscommunication were noticed.
4	Coping with Covid-19 from the Capability Perspective: A View from a Developing Country	Osmani et al., 2020	**Study Area and Sample:** Bangladesh	Review	• An extensive system of public health support was needed to relax the shutdown, size and duration of economic support depends crucially on the availability of public health support. • Testing capacity for COVID-19 needed to be increased; highly effective organisational framework needed.
5	Challenges of Testing COVID-19 Cases in Bangladesh	Rahaman et al., 2020	**Study Area and Sample:** Bangladesh; Qualitative, quantitative and GIS data	Mixed method	• Negligible number of available beds; testing centre allocation over the country was not proportionately distributed to the population. • Significant scarcity of testing centres testing centres per thousand people, accelerated by the enforced immobility and lockdown activities in the country
6	Moral distress among healthcare providers and mistrust among patients during COVID-19 in Bangladesh	Hossain 2020	**Study Area and Sample:** Bangladesh	Review	• lack of resources, combined with mismanagement and corruption, severely challenges the country's healthcare system, health sector needs remain unattended • COVID-19 unsettled the already fragile 7 healthcare system of Bangladesh, moreover the lockdown negatively impacted the economy; misleading information and people's mistrust on health system spilt over to the health care providers as well.
7	Seeking an ethical theory for the COVID-19 pandemic outbreak with special reference to Bangladesh's law and policy	Bhuiyan 2020	**Study Area and Sample:** Bangladesh	Report	• In the pandemic management policy paper adopted by the Bangladesh Government's health sector in March 2020, no emphasis on the implementation was seen failing to incorporate bioethical strategies to handle people's lives • Issue raised on the scarcity of resources and the fair distribution of emergency medicines, medical equipment
8	COVID-19 Pandemic Is About More than Health: A State of Governance Challenges in Bangladesh	Uddin 2021	**Study Area and Sample:** Comparison between Brunei, Cambodia, Sri Lanka, Taiwan, Thailand, Vietnam and Bangladesh	Qualitative study	• Lack of effective governance and poor management of the COVID-19 since the governance structure has experienced extensive corruption, political unaccountability, poor public involvement etc. • Although GoB had ample amount of time, it was reluctant to respond to the declaration of the PHEIC and was unresponsive to management and preparedness of the pandemic and failed to manage public health.
9	The moral and political economy of the pandemic in Bangladesh: Weak states and strong societies during Covid-19	Ali et al., 2021	**Study Area and Sample:** 6 locations (Rural haor, Rural coastal and Peri-urban area) in six districts, Bangladesh **Time frame:** 2^nd^ week of April; 3^rd^ & 4^th^ week, May 2020	Case study	• The govt enforced administrative orders without any public announcement, although it failed to manage lockdown. • Although promises were made, corruption in relief distribution created uncertainties; lack of fairness and effectiveness in response • In peri-urban and rural sites, people who didn't need relief seemed to have benefitted more
10	Women's Knowledge, Attitude and Perceptions Toward COVID-19 in Lower-Middle-Income Countries: A Representative Cross-Sectional Study in Bangladesh	Anwar et al., 2020	**Study Area and Sample:** Among 1869 adult women**;** 61 districts **Time frame:** Initial weeks of lockdown	Cross-sectional survey	• Lack of effort in educating women, especially older women • Significant proportion of participants thought that governments efforts in controlling the pandemic were inadequate • More than 50% thought the government was not transparent on COVID-19 related information.
11	Healthcare Crisis in Bangladesh during the COVID-19 Pandemic	Al-Zaman 2020	**Study Area and Sample:** Bangladesh	Perspective piece	• Health sector corruption continued even during the COVID-19 pandemic • Limited number of facilities for COVID-19 testing; at the same time crisis was created due to corruption in the process • Corruption and mismanagement were present in getting medical facilities, treatment; fake medical equipment, PPE were supplied • Health related uncertainty, lack of reliable information, rumours was related with the disorganised health sector

## 3 Results

A total of 11 articles on COVID-19 ‘governance’ were selected for review (Table 3). Of these, two were cross-sectional surveys; two were qualitative studies; two were reviews; two were perspective pieces, and one each of case study, report and a mixed-method study. The review findings are described below under the following thematic headings.

### 3.1 Quarantine measures: from enforcement to relaxation

From the very beginning, governance problem was prominent in managing the lockdown to limit the spread of the virus, characterised by ‘confusion, incoherence, and reversal’ [20]. In the early weeks, the health system displayed poor preparedness in tackling the returnees from Italy (and other countries) by institutional ‘quarantine’ in the airports or, later, in the homes [[20], [21], [22]]. The expatriates at the airports revolted against the poor arrangement of quarantine facilities, and the authorities had to succumb and release them. Similarly, unsupervised home quarantine was ignored by people and was largely ineffective. The first lockdown of 66 days (from 26 March to May 30, 2020, starting with a ten-day ‘national holiday’ and extended seven times) started with a robust enforcement regime. People initially supported the stringent measures as they panicked from the unknown virus and the consequent disease. Over time, when the economic condition of the poor worsened and the government failed to provide subsistence relief to the poor and marginalised, coercive lockdown gave way to exceptions [20]. Excuses such as permitting the “boro rice harvest,” “mosques to host congregations with social distancing rules,” “restaurants to sell *iftar* foods,” “garments factories and shops to reopen” etc. were put forward to loosen the ‘lockdown’ restrictions. Researchers came forward with advice to strategically tackle both lives and livelihood simultaneously and reinforce ‘safety net’ entitlements to overcome the situation [23].

### 3.2 Testing for COVID-19: limited to a single institution initially

The primary tool to fight COVID-19, i.e. facilities for testing the presence of SARS-CoV-2, was restricted to a single government institution until the end of March 2020, leaving many districts and sub-districts without any such facility in the early weeks [21,24]. Many facilities with test kits could not implement a standardised test protocol due to the absence of biosafety level 2 labs or a shortage of trained personnel. The number of test labs and tests performed was meagre compared to the size of the population in the country (13 tests per million) [25] and not proportionally distributed geographically. This shortage of tests resulted in fewer reported cases and left the decision-makers without an efficient epidemiological tool to pursue an evidence-based Governance of COVID-19 [25]. The number of test labs slowly expanded over time, however.

### 3.3 All-pervading corruption: dent in trust in health systems

What shocked the nation was the continuation of the all-pervading health sector corruption even during the ongoing pandemic [10]. Prominent among these and frequently reported in media, were corruption related to the procurement of personal protective equipment, masks and other supplies; fake COVID-19 testing and report (e.g., Regent Hospital and JKG Health Care scams); five hundred physicians' food and living costs for one month; and the relief regime for the poor and the disadvantaged. These caused many unnecessary deaths, including doctors and nurses, and raised the cost of care for COVID-19 patients [26].

The level of corruption during the pandemic made a dent in people's trust in the health system, especially its management [12], which also spilt over to the healthcare providers (especially doctors and nurses) [27]. This hampered government's ability to act ‘decisively’ and ‘transparently,’ e.g., in enforcing ‘lockdown’ measures when needed. This lack of “state capacity to make and enforce policy,” e.g., related to lockdowns, was argued by some authors from a political economy perspective as a reflection of the need to demonstrate outcome performance in the face of questionable political legitimacy of the government [20].

### 3.4 Improving governance: suggested measures

The stakeholders made various suggestions, including facilitation of local governance, data-driven governance and emphasising the moral aspects of governance to counter the problems observed with COVID-19 governance. By activating and mobilising the local government bodies, the community was found to effectively engage in COVID-19 mitigation measures, including the delivery of essential healthcare services [28]. For the latter, ICT tools and local governance digitisation increased accountability and transparency by connecting more people to the decision-making process.

A four-tier bio-ethical ‘Pandemic Outbreak Disaster Management Model (PODM)' was proposed incorporating issues concerning ‘life, living beings, interests of victims, food safety, necessary medical equipment, and medicine,’ over and above that described in the government's National Preparedness and Response Plan for COVID-19 [29]. The proposed tiers consisted of i) critical assessment of the current scenario to develop a response; ii) understanding the global experiences of pandemic impacts and using it for the action plan; iii) helping people recognise how they should interact with the consequences of a pandemic situation; iv) strengthened techniques and capacity to bring life back to ‘normal’ post-pandemic.

## 4 Discussion

This study presents critical reflections on Bangladesh Government's COVID-19 governance response through a review of selected papers on the topic (n = 11), followed by expert deliberations on the review findings. Findings reveal a lack of governance capability to mount a quick, effective and efficient response, such as lockdown and testing, that is inclusive and comprehensive [22]. The findings are discussed below with implications for future epidemic/pandemic preparation in the context of LMICs like Bnagladesh.

The governance challenges against COVID-19 in Bangladesh came out starkly in the articles reviewed [[20], [21], [22]]. Experiences from a few Asian countries (Brunei, Cambodia, Sri Lanka, Taiwan, Thailand and Vietnam) ‘beating’ COVID-19 reiterated the importance of an effective and efficient governance mechanism that Bangladesh lacked [22]. For effective and inclusive governance of a health emergency of the magnitude of COVID-19 pandemic, a ‘whole of government’ and a ‘whole of society’ approach is essential which was lacking in Bangladesh, at least in the early months [[30], [31], [32]].

The pandemic was an eye-opener to the fundamental shortfalls of the Bangladesh health systems such as shortage of health workforce of all categories and at all levels, essential diagnostics, personal safety gear for the frontline healthworkers, and medical oxygen supply and intensive care units (ICUs). This is not surprising, and consistent with what was observed in the latest Bangladesh Health Facility Survey (2017). The survey found only 28% of the static health facilities having all the six basic equipment (stethoscope, thermometer, blood pressure machine, adult weight scale, child or infant scale, and light source) which are essential for providing services of a minimal quality [33].

In the early months, lockdown and other non-pharmaceutical measures were the only available tools to contain the spread of the infection. The lockdown measures give health systems breathing space to prepare for the surge in COVID-19 suspects and patients. However, due to resource constraints (e.g., shortage of skilled health care workers, leakage of financial resources due to health sector corruption, lack of lab and other technical capacity), many low and middle-income countries (LMICs) could not prepare the health systems in time for lockdown, and Bangladesh was no exception [20,34,35]. Lockdown measures in LMICs should be contextualised for local conditions, beneficial for the concerned population, and prevent the need for re-imposing lockdown [36]. In these countries, a localised lockdown of the hotspots, combined with disease surveillance and other non-pharmaceutical measures should be implemented [35]. For this to happen, science needs to be prioritised over politics, actively engage the communities in COVID-19 containment activities, and recruit and train more health care workers to work at the front lines.

Corruption in the health sector of Bangladesh is phenomenal [37]. The COVID-19 pandemic not only unmasked the weaknesses of the health system in this regard, but also “created new opportunities for corruption” [10,38]. This trend of continued corruption in the times of pandemic has been observed globally as well [[39], [40], [41]]. Various strategies are suggested to reiterate that anticorruption must remain a priority even during pandemic like COVID-19 [41]. These include, but not limited to, recruitment of appropriately skilled staff, positioning public health experts in command (“put science before politics”) [42], and a gender perspective to ensure that anticorruption measures “do not further marginalise or disadvantage women and other vulnerable and marginalised groups” [41].

Globally, “populist” governments did a lousy job of responding to COVID-19 due to inappropriate policy responses, downplaying the gravity of the pandemic and putting politics over science [43], as was also observed in case of Bangladesh. Anti-intellectualism (generalised distrust of experts and intellectuals) among the masses at large also contributes to this kind of “populist” governance because people usually comply with antipandemic measures when the information comes from a source they trust [44].

The importance of ‘good governance’ for a successful COVID-19 response cannot be overemphasised. Globally, argument is made to turn the COVID-19 health crisis into an opportunity to prepare for the next epidemic/pandemic [36,45,46]. In Bangladesh, the leadership must prepare for such crisis and follow ethical principles to overcome various forms of incompetencies in the system. These include mismanagement of resources allocated (e.g., for COVID-19), failure to harmonise coordination among government's different agencies (e.g., relevant to COVID-19 response), and resilience to deliver services (e.g., for COVID- 19 cases) without compromising essential services, e.g., as has been observed in India [47].

### 4.1 Strengths and weaknesses

The findings from the reviewed papers were further shared and discussed with experts in the field which strengthened the validity of the information. We restricted the search of the papers available within the repository and might have missed papers on the topic, especially related to the later part of the pandemic, whose includion would have affected the analysis.

## 5 Conclusions

Bangladesh's COVID-19 response in the early months was characterised by slow, delayed and ambiguous measures which reflected poorly on its governance [48]. Governance gaps in areas such as instituting screening and lockdown measures, prioritising safety and security of the frontline workers to preserve the workforce, COVID-19 testing, quarantine (suspects) and isolation (cases), and logistics and procurement was phenomenal. However, over time, the GoB laid down required actions and services for mitigation of the pandemic impact on the country, though the stewardship functions were not seamless. Diagnostic and case management services gained strength after some initial faltering. Continued shortage of all kinds of healthworkers, poor capacity of the health facilities to cater to the COVID-19 suspects and cases, particularly outside the major metropolises, and constraints in resources and logistics were some of the critical factors limiting government's COVID-19 responses. The scarcity of governance related articles indicate the need for more focused research in this area, so that substantial contributions can be made through indentifying gaps in policies and regulations.

## Ethics approval and consent to participate

Not applicable.

## Patient consent for publication

Not applicable.

## Availability of data and materials

All data generated and analysed during this study are included in this published aeticle.

## Funding

This research did not receive any specific grant from funding agencies in the public, commercial, or not-for-profit sectors.

## Declaration of competing interest

The author(s) declare that there is no potential conflict of interest.
